# Vision-related quality-of-life in pediatric primary brain tumor patients

**DOI:** 10.1007/s11060-021-03835-2

**Published:** 2021-08-30

**Authors:** Jason H. Peragallo, Beau B. Bruce, Caroline Vasseneix, Supharat Jariyakosol, Anna J. Janss, Nancy J. Newman, Valérie Biousse

**Affiliations:** 1 Departments of Ophthalmology, Emory University School of Medicine, Atlanta, GA; 2 Departments of Pediatrics, Emory University School of Medicine, Atlanta, GA; 3 Departments of Epidemiology, Emory University School of Medicine, Atlanta, GA; 4 Departments of Neurology, Emory University School of Medicine, Atlanta, GA; 5 Aflac Cancer & Blood Disorders Center, Children’s Healthcare of Atlanta, Emory University School of Medicine, Atlanta, GA; 6 Departments of Neurological Surgery, Emory University School of Medicine, Atlanta, GA

**Keywords:** quality-of-life, vision, pediatric, primary brain tumor, neuro-ophthalmology

## Abstract

**Purpose:**

Brain tumors are the leading cause of death from childhood cancer. Although overall survival has improved due to earlier detection, better therapies, and improved surveillance, visual dysfunction and impaired vision-related quality-of-life (VR-QOL) are often unrecognized in children. This project investigated VR-QOL in pediatric brain tumor patients.

**Methods:**

We evaluated visual impairment and quality-of-life (QOL) in a quality improvement project at one tertiary care center. Patients ≤18, greater than 6 months from diagnosis of brain tumor, excluding intrinsic anterior visual pathway tumors, underwent standardized neuro-ophthalmologic examination. Health-related QOL (HR-QOL) [PedsQL Brain Tumor Module] and VR-QOL questionnaires [CVFQ (Children’s Visual Function Questionnaire) in children ˂8, and EYE-Q in children 8–18] were obtained from patients and parents.

**Results:**

Among 77 patients, craniopharyngiomas (n=16, 21%) and astrocytomas (n=15, 20%) were the most common tumors. Among 44/77 (57%) visually impaired children, 7 (16%) were legally blind. Eye-Q median score was 3.40 (interquartile range 3.00–3.75), worse than average scores for normal children. Eye-Q score decreased 0.12 with every 0.1 increase in logMAR visual acuity [p<0.001]. Patients who were legally blind had a significantly lower Eye-Q score than those who were not (0.70 vs. 3.44 [p<0.001]). Cognitive HR-QOL scores decreased 1.3 for every 0.1 increase in logMAR visual acuity [p=0.02].

**Conclusions:**

Pediatric brain tumor patients’ vision, HR-QOL, and VR-QOL were often severely affected even when tumors were considered cured. Visual acuity and legal blindness correlated with VR-QOL. Systematic neuro-ophthalmologic examinations in pediatric primary brain tumor patients are necessary to facilitate early preventative and corrective ophthalmologic interventions.

## Introduction

Primary central nervous system (CNS) malignancies are the most common cause of cancer, as well as the most common cause of death from cancer, in the pediatric population [1], with an increasing overall incidence in the United States [2]. An estimated 4,630 children and young adults will be diagnosed with brain and CNS tumors in 2021 [1]. Management of children with brain malignancies is complex, involving a multi-disciplinary team approach including neuro-oncology, neurology, neurosurgery, radiation oncology, endocrinology, neuroradiology, neuropsychology, and neuro-ophthalmology. Survival is improving through new treatment modalities, better disease surveillance, and improved surgical and radiotherapy techniques. As survival has improved, emphasis on post-treatment quality-of-life (QOL) has increased [3]. Indeed, future education opportunities, independence, employment opportunities, and even driving ability may be affected and lead to decreased QOL.

Despite common involvement of cerebral visual pathways by brain tumors, ophthalmologic evaluations are not standard of care for all brain tumor patients and less than half are referred for ophthalmic evaluations [4,5]. Reasons cited for not obtaining ophthalmology evaluation early in the management of a child with a primary brain tumor include the difficulty of examining a young child, focus on treating the primary disease, lack of access to a pediatric ophthalmologist or neuro-ophthalmologist, medical providers’ lack of awareness of the risk of vision loss, and lack of complaints by a child who unknowingly has a visual deficit [4]. Children notice decreased vision less often and may not be able to verbalize or explain their symptoms.

Several studies of how brain tumors and their treatment affects health-related QOL (HR-QOL) in children have been performed [4]. However, little is known about how HR-QOL is specifically affected by visual impairment in this population. To our knowledge, no studies have specifically addressed VR-QOL of pediatric primary brain tumor (PBT) patients who have tumors that are not intrinsic to the visual pathways, and therefore not obviously expected to be visually impaired. To improve care and referral frequency at our institution, we aimed in a quality improvement project to evaluate how changes in vision in PBT patients affect QOL through evaluation with vision-related QOL (VR-QOL) surveys.

## Methods

This quality improvement project was evaluated by the Emory University Institutional Review Board and was found to be exempt from formal review. The project conformed to the requirements of the Declaration of Helsinki and the United States Health Insurance Portability and Accountability Act.

Consecutive children diagnosed with primary brain tumors not intrinsic to the primary visual pathways (new or established patients) who were evaluated at the AFLAC Cancer and Blood Disorders Center at Children’s Healthcare of Atlanta between June 2014 and August 2018 were referred to the pediatric neuro-ophthalmology unit at Emory University for systematic evaluation as part of their standard of care. Patients with intrinsic primary tumors of the anterior visual pathways (i.e., optic pathway gliomas) were not included as ophthalmologic evaluations at regular intervals to monitor for deterioration of vision are considered standard of care for these patients, and have previously been extensively studied [6]. This quality improvement project was initiated to improve, and stress the importance of, the referral process from neuro-oncology to the ophthalmology service as we were aware of many patients who were not evaluated in a timely fashion after their initial diagnosis and treatment was complete for various reasons (such as lack of access, lack of providers, perceived limited usefulness of evaluation). To reduce bias of QOL scores by recent diagnosis and treatment of the underlying tumor, patients included in the project were at least 6 months post initial brain tumor diagnosis. Basic demographic data was collected, including age, sex, and race. Oncologic data collected included date of diagnosis, tumor type, location (frontal, parietal, temporal, occipital, thalamic, sellar/suprasellar, pineal, brainstem/fourth ventricle), and treatment modalities.

All children included in this project underwent standard age-appropriate neuro-ophthalmic evaluations, including best corrected visual acuities (VA) in each eye (Snellen, LEA symbols, HOTV, or central, steady, maintained (CSM) method, based on age and cooperation), refraction, color vision evaluation with Ishihara plates, pupillary examination, sensorimotor examination, confrontation visual field (VF) testing, slit lamp examination, and dilated funduscopic examination. When possible, formal VF testing was performed on an automated static perimeter (Humphrey 24–2 SITA-Fast protocol) or manual kinetic perimetry (Goldmann), based on age and cooperation. Fundus photography and optical coherence tomography (OCT) of the retinal nerve fiber layer (RNFL) were obtained on all patients who could cooperate (Cirrus HD-OCT, Carl Zeiss Meditec AG, Jena, Germany). Legal blindness is defined in the United States as best corrected VA in the better eye of 20/200 or worse, and/or VF in the better eye of 20 degrees or less. For this project, we defined visual impairment as best corrected VA of 20/40 or worse in the better eye (or 3 lines worse than appropriate vision for age) [7], complete loss of vision in one eye, or a bitemporal or homonymous hemianopic VF defect. For the purposes of analysis, if patients were unable to cooperate with a form of VA testing that would produce a numerical value, these patients were not considered visually impaired from a VA standpoint unless they were determined to be light perception or no light perception in that eye. When VA was classified as impaired compared to normal, the underlying cause was classified as resulting from direct tumor or treatment involvement, papilledema or its sequelae, amblyopia, both direct tumor involvement and papilledema, or corneal complications. When VF defects led to a patient to meeting criteria for visual impairment, the underlying cause of the field defect was classified as resulting from direct tumor or treatment involvement, papilledema, or from a combination of both.

Patients and parents were asked to complete QOL questionnaires based on age at the time of the neuro-ophthalmology visit. The questionnaires were completed on pen and paper by the parents and their children in the ophthalmology clinic during their visit. For children unable to read and complete the forms, the parents read the questions to the children and filled in the response. Parents of children 3–7 years old were asked to complete the age appropriate PedsQL Brain Tumor Module Parent Reports for Toddlers [8] and Children’s Visual Function Questionnaire (CVFQ) [9,10]. Children 5–7 years old were additionally asked to complete the PedsQL Brain Tumor Module Young Child Report [8]. Children 8–18 years old were asked to complete the age appropriate PedsQL Brain Tumor Module Reports and Eye-Q Questionnaire [11], and their parents were asked to complete the age appropriate PedsQL Brain Tumor Module Parent Reports [8]. The PedsQL brain tumor module is a validated HR-QOL questionnaire utilizing a 3-point or 5-point Likert scale dependent upon age, ranging from a score of 0 to a score of 4, with higher scores indicating fewer problems. The PedsQL brain tumor module assesses the dimensions of cognitive problems, pain and hurt, movement and balance, procedural anxiety, nausea, and worry. The CVFQ is a validated VR-QOL questionnaire utilizing a 5-point Likert scale dependent upon age, ranging from a score of 0 to 1, with higher scores indicating fewer problems. The CVFQ assesses the dimensions of general health, general vision, competence, personality, family impact, and treatment. The Eye-Q is a validated VR-QOL questionnaire for children 8–18 years of age consisting of 23–36 questions, depending on age, with 3 additional questions included regarding driving for those 16 years of age and older. Self-reported responses utilize a 5-point Likert scale evaluating tasks at near, distance, color, night vision, functionality, and photosensitivity. Eye-Q score range is from 0–4, with higher scores indicating less problems. Missing QOL questionnaire data was not scored and the questionnaire score denominator (number of questions) was adjusted accordingly. Additional questions about the need for low vision aids, special assistance at school, and driving status (if 15 years or older) were asked [Appendix 1].

For purposes of analyses Snellen VA was converted to logMAR VA (-log(Snellen)), where logMAR 0 corresponds to Snellen acuity of 20/20, and logMAR 1.0 corresponds to Snellen acuity 20/200. Statistical analyses were performed using the R:A language and environment for statistical computing (R Foundation for Statistical Computing, http://www.R-project.org). Medians were reported for continuous data; percentages were reported for categorical data. Medians were compared using the Mann-Whitney *U* test, and proportions were compared using χ^2^ or Fisher exact tests, as appropriate. Logistic regression was used to determine the association of age with success and multiple procedures. Linear regression was used to determine the association of QOL measures with continuous covariates, e.g., visual acuity and RNFL thickness. Two-tailed *p* values <0.05 were considered statistically significant.

## Results

### Vision outcomes

This quality improvement project evaluated 77 patients. Median age at diagnosis was 6.5 years (IQR 3.7–11, range 0.2–17.1). The most common tumor pathologies included were craniopharyngioma (n=16, 21%), followed by astrocytoma (n=15, 20%) [Table 1]. Of the 77 patients, seven (9%) met the criteria for legal blindness [Table 2]. A total of 44 (57%) patients were visually impaired (including those who were legally blind) [Table 2]. Visual impairment from VA loss was present in 22 patients (29%). Of the 44 patients with visual impairment, 24 (55%) were receiving assistance at school and 17 (39%) had assistive visual aids. Total number of patients receiving assistance at school was 44 (57%) and total number of patients using assistive visual aids was 44 (57%).

### Vision loss and Quality of Life vs. underlying cause

Underlying cause for decreased VA was associated with a significant change in Eye-Q score (p=0.033) [Table 2]. Decreased VF meeting our criteria for visual impairment was present in 41 patients (53%), and underlying cause was not associated with Eye-Q score [Table 2]. Optic nerve atrophy was noted unilaterally in 2 patients (3%) and bilaterally in 27 patients (35%) [Table 2]. Underlying cause of optic nerve atrophy (direct tumor or treatment involvement, previous papilledema, or a combination of tumor or treatment involvement and papilledema) was associated with a statistically significant difference in Eye-Q score (p=0.03), with a combination of underlying causes of optic atrophy leading to worse Eye-Q scores [Table 2]. Strabismus was present in 25 patients (32%). Underlying cause of strabismus was not found to be associated with Eye-Q score [Table 2]. Two patients (3%) had ocular motor cranial nerve palsies.

### VR-QOL and HR-QOL vs. Vision outcomes

**Fig. 1:**
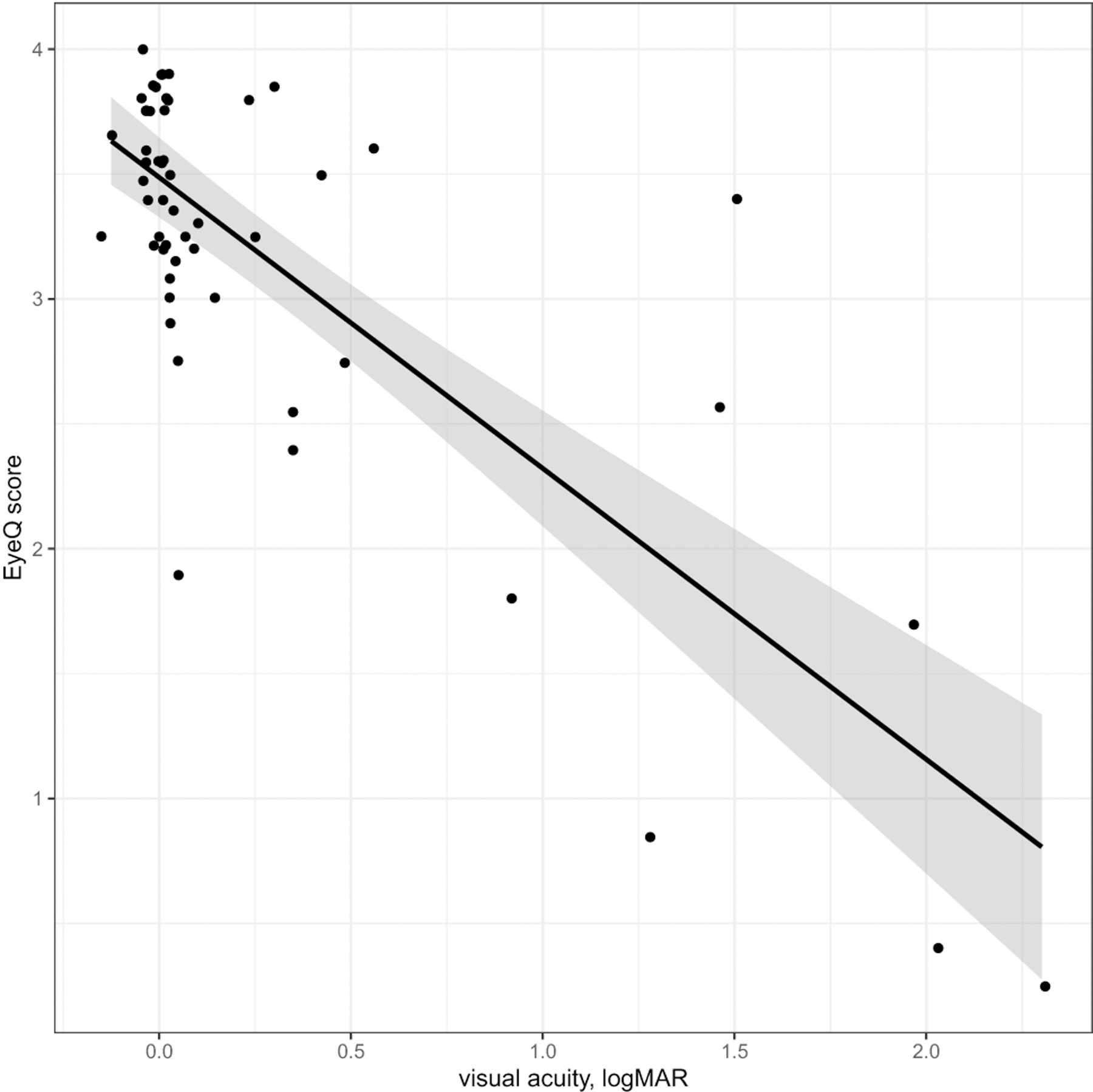
logMAR visual acuity vs. Eye-Q Score (points jittered to reduce overlap) – Linear regression showed that Eye-Q score decreased by 0.12 for every 0.1 increase in logMAR visual acuity (worsening vision) [p<0.001]. LogMAR visual acuities = (-log(Snellen)), where logMAR 0 is equivalent to 20/20, logMAR 0.3 is equivalent to 20/40, and logMAR 1.0 is equivalent to 20/200

**Fig. 2:**
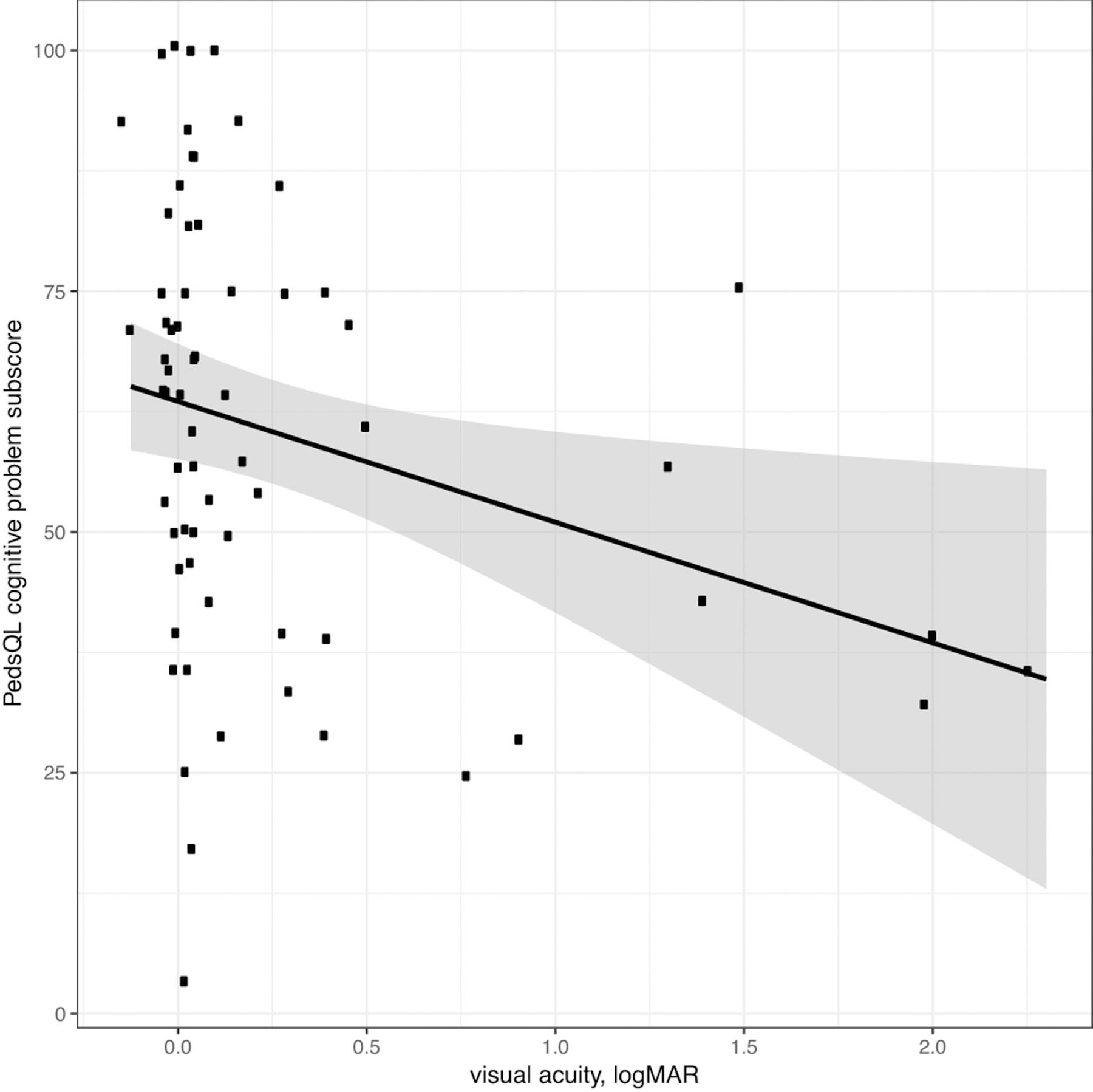
logMAR visual acuity vs. PedsQL Cognitive Problem subscore (patient) (points jittered to reduce overlap) – Linear regression showed that PedsQL Cognitive Problem subscore decreased by 0.13 for every 0.1 increase in logMAR visual acuity [p=0.02]. LogMAR visual acuities (-log(Snellen))

**Fig. 3:**
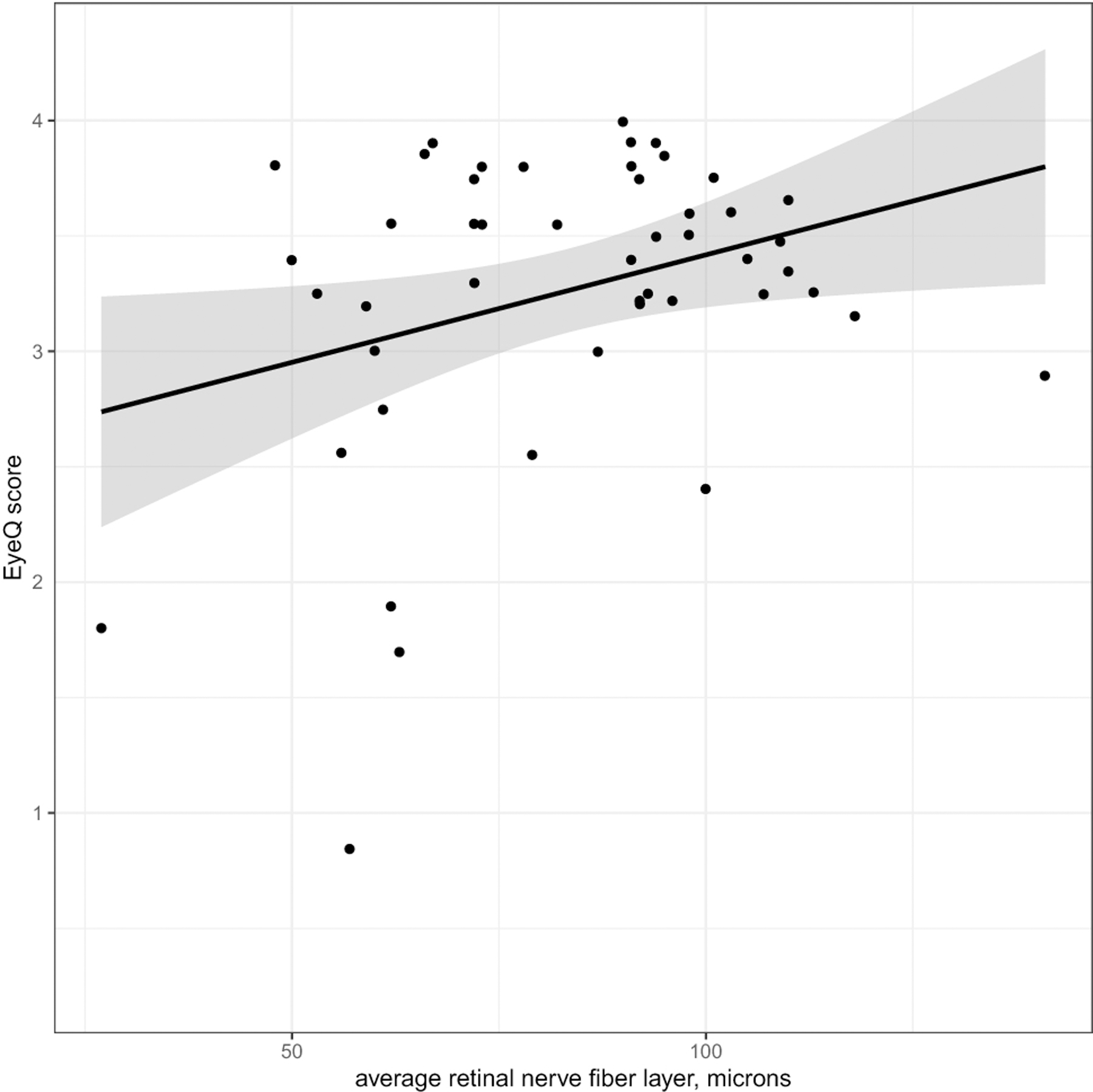
RNFL vs. Eye-Q score – (points jittered to reduce overlap) – Linear regression showed that Eye-Q score decreased with decreasing retinal nerve fiber layer thickness measured by optical coherence tomography [p=0.03]

**Fig. 4:**
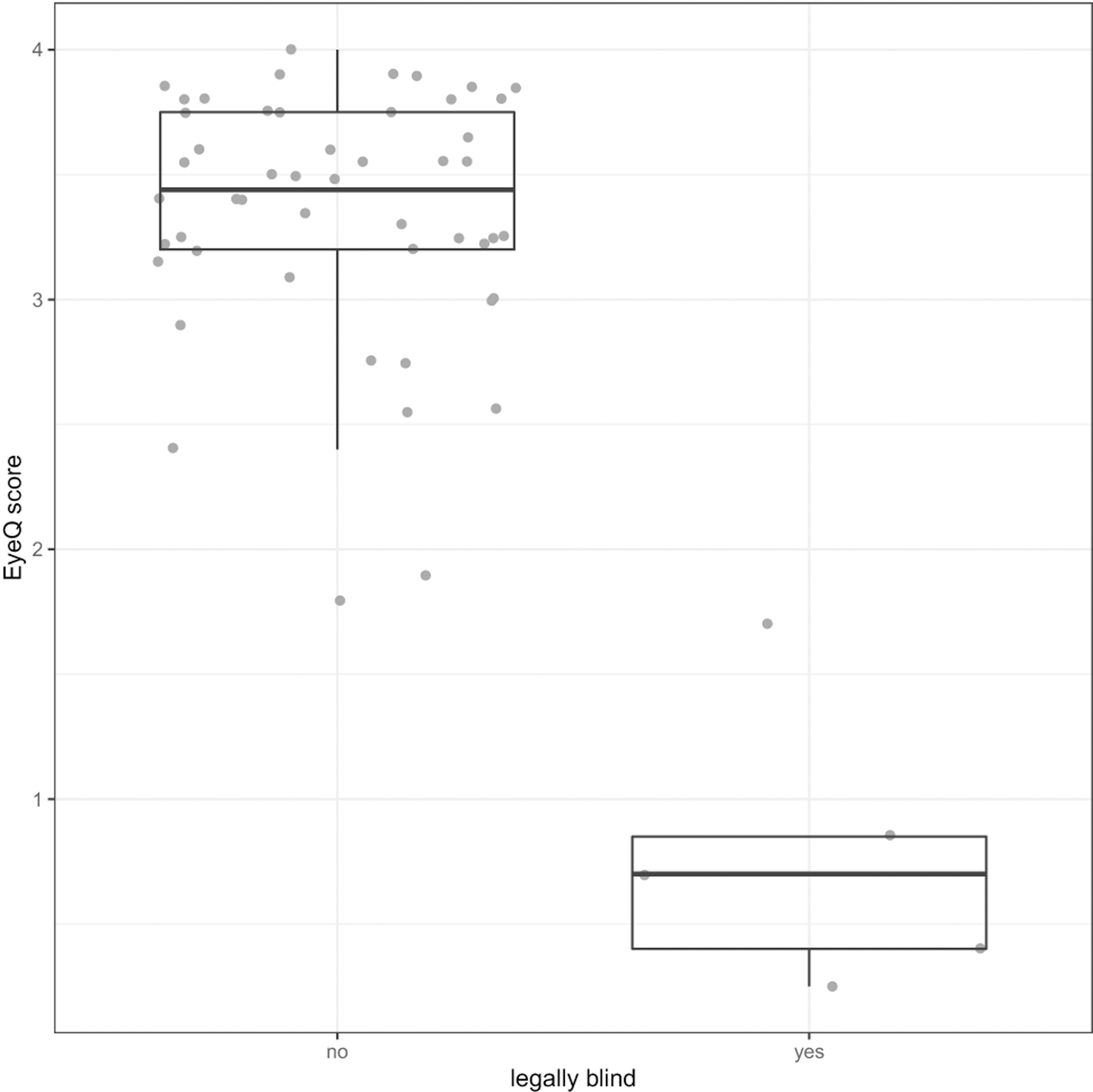
Box plot of Legal blindness status vs. Eye-Q Score with jittered individual observations –- Patients who were legally blind had an Eye-Q score on average of 0.7 compared to 3.43 for those who were not legally blind [p<0.001]. Legal blindness defined as Snellen visual acuity of 20/200 or less in the better seeing eye or remaining visual field in better seeing eye of less than 20 degrees

Average Eye-Q VR-QOL score was 3.40. Patients with worse VA had a lower Eye-Q score, with a 0.1 increase in logMAR VA (decrease in Snellen VA) corresponding to a decrease in Eye-Q score of 0.12 (p<0.001) [Figure 1]. Worse VA was also associated with lower HR-QOL cognitive scores, with a 0.1 logMAR VA worsening corresponding to a 1.3-point decrease in HR-QOL cognitive scale (p=0.018) [Figure 2]. A trend toward increased worry about the tumor being associated with VA loss was observed for both patients and proxies (p=0.06, p=0.06). Legally blind patients had a significantly lower HR-QOL worry score compared to those who were not legally blind (p=0.016). Patients with thinner average RNFL (corresponding to optic nerve atrophy) on OCT also had lower VR-QOL scores (p=0.03) [Figure 3]. Patients who met criteria for visual impairment had a lower VR-QOL score of 3.25 compared those not visually impaired at 3.55 (p=0.05). Twenty-two patients met our criteria for decreased VA; their mean VR-QOL score was 2.55, compared to 3.55 in patients with better VA (p<0.01). Legally blind patients had a significantly lower VR-QOL mean score compared to those who were not legally blind of 0.7 vs. 3.43 (p<0.001) [Figure 4]. Comparisons of VR-QOL scores by age were not significant. Eight patients were less than 8 years old at examination, and the average CVFQ score for these patients was 0.72. The small number of patients limited further analysis in this age group.

### VR-QOL and HR-QOL Outcomes vs. Tumor characteristics and interventions

Surgical resection was performed in 57 patients (74%), chemotherapy in 43 (56%), and radiation in 39 (51%). An Ommaya reservoir was placed in 6 patients (8%). A total of 5 patients (6%), including those with prolactinomas, were treated with hormonal therapy. Underlying tumor type, individual treatment modality, or combination of treatment modalities was not associated with decreased visual function, HR-QOL, or VR-QOL. When analyzed by specific location, and by more generalized supratentorial versus infratentorial location, tumor location was not associated with decreased visual function, HR-QOL or VR-QOL.

## Discussion

We found that pediatric patients with primary brain neoplasms not intrinsic to the anterior visual pathways had significant ophthalmologic morbidity associated with their underlying disease. Indeed, 44 (57%) patients met our criteria for being visually impaired, compared with 10.6% in a study of patients with posterior fossa tumors and 66% among patients with craniopharyngiomas [12,13]. Seven (9%) patients met criteria for legal blindness, which is similar to the rate found for craniopharyngiomas (10%) [13]. In patients with posterior fossa brain tumors, the rate of VA meeting criteria for legal blindness ranged from 0–7.4%, (although the 7.4% number includes 3/9 total patients with “good” VA in one eye) [12,14].

We found VF defects in 53% of patients. A previous study which included intrinsic visual pathway tumors found visual field defects in 26% [5]. A study including only children with craniopharyngioma found visual field defects in 60% [15]. When a VF defect is a homonymous or bitemporal hemianopia, the consequences for QOL, including aspects of education, employment, and independence, can be devastating because these visual deficits often preclude the legal ability to drive. Indeed, one study found that only 57% of PBT patients were able to drive [16]. One previous analysis of 36 pediatric patients with homonymous hemianopias found that brain tumors caused 39% of the hemianopias [17]. Another retrospective analysis of homonymous hemianopias in 86 children found that 27% were due to brain tumors [18]. These VF defects may go undetected in children with PBTs due to lack of awareness on the part of the child, combined with the treatment team focusing on the primary brain tumor and its management, possibly neglecting the need to have the child evaluated by an ophthalmologist to detect vision loss. In fact, a systematic neuro-ophthalmic examination of patients who had already been evaluated and treated for PBTs found a rate of previously undetected VF defects of 15.2% [19].

An important characteristic of the visual system in childhood is the continuous development during early years, placing children at particular risk for developing irreversible visual loss from amblyopia. When detected early, amblyopia can be treated, and permanent visual loss can be prevented. Children with brain neoplasms are at higher risk for developing ptosis, strabismus, and cranial nerve palsies, which can all lead to amblyopia. Liu et al. found a rate of amblyopia from any cause in their population of PBT patients of 38% [5]. Prompt ophthalmologic evaluation is therefore necessary in children with brain tumors who exhibit these signs.

Intrinsic anterior visual pathway tumors, such as optic pathway gliomas, cause vision loss most commonly from direct compression and infiltration. These patients are usually monitored serially for visual compromise by an eye care provider. However, patients with neoplasms arising from other locations in the brain are less likely to undergo ophthalmologic evaluations. In brain tumors not arising from the primary visual pathways, there are multiple mechanisms for potential vision loss. Firstly, the tumor, or mass effect from surrounding edema, can directly compress the intracranial visual pathways such as optic nerves, optic chiasm, lateral geniculate ganglia, optic radiations, or occipital lobes. A second mechanism for vision loss is papilledema from obstructive hydrocephalus or increased intracranial volume [20]. Amblyopia is a third mechanism for vision loss specific to children. We found the most common causes of decreased vision were tumor involvement or treatment effects, followed closely by amblyopia [Table 2].

Chronic papilledema can lead to permanent, severe vision loss and optic atrophy. VA and VF loss were attributed at least in part to previous papilledema in 27% and 31% of our patients, respectively. In a retrospective analysis of patients who presented to the emergency department who were diagnosed with brain tumors, 20% of the patients presented with vision changes and 13% of all of the brain tumor patients had papilledema at presentation [21]. An evaluation of children who were diagnosed with posterior fossa tumors found that 39.7% had papilledema among the 56% of the cohort who underwent funduscopic examination [14]. Among pediatric patients with posterior fossa tumors, 17.2% had poor VA (worse than 20/40 in at least one eye) after treatment of the underlying lesions, likely due to the consequences of hydrocephalus and papilledema [14].

Decreased vision and diplopia often seen in brain tumor patients can adversely affect QOL for the child and their family. Children who are visually impaired have been shown to have 35.6% lower QOL scores compared to healthy age-matched controls using the Low Vision Quality of life questionnaire [22]. Other studies have found that disorders of the visual pathways, involving anywhere from the optic nerve to the visual cortex, lead to lower QOL scores than disease of the eyes themselves [23].

Studies evaluating PBT treatments have used HR-QOL questionnaires extensively to measure QOL before, during, and after treatment. We previously reviewed the literature regarding the frequency of visual impairment in PBT patients in studies evaluating the effect of PBTs on HR-QOL [4]. We chose the PedsQL HR-QOL questionnaire due to its frequent use in the pediatric literature and its specific brain tumor module. For VR-QOL, we chose to use the CVFQ for children 7 years of age and younger for its previous validation and use in prior studies of VR-QOL in optic pathway gliomas [24], as well as its recommendation for use by the Response Evaluation in Neurofibromatosis and Schwannomatosis (REiNS) International Collaboration Visual Outcomes Committee [25], and the Eye-Q for children 8–18 years of age due to its previous validation as well as its inclusion of questions regarding driving [11]. Compared to normal patients in a previous unrelated study, our patients overall had lower VR-QOL scores on the Eye-Q questionnaire (3.40 vs. 3.71) [11]. Eye-Q scores have been found to correlate to degree of visual impairment, with normal vision in the better seeing eye corresponding to a value of 3.65±0.37, mild visual impairment of 2.99±0.62, moderate visual impairment of 2.83±0.48, and severe visual impairment of 2.16±1.14 [11]. A new VR-QOL validated questionnaire, the PedEyeQ, was not available to us during this project period, but may be useful in future studies [26].

In the only study to our knowledge of PBTs to date evaluating VR-QOL, Avery and Hardy found that patients with optic pathway gliomas and vision loss had lower VR-QOL scores on the Competence and Family Impact domains compared to patients with optic pathway gliomas and normal or borderline vision as measured by the CVFQ, and also had lower Competence and Personality scores if both eyes had impaired vision compared to those with vision affected in one or neither eye [24]. VR-QOL has been evaluated in adult patients with pituitary adenomas, and has been found to be decreased for this population (using the VFQ-25 questionnaire) and correlated with the VF deficit in the better seeing eye and length of time of ocular symptoms [27]. In adults undergoing transsphenoidal surgery for pituitary adenomas, VR-QOL improved, particularly for patients with worse VF defects and VR-QOL scores prior to surgery [28]. However, we were unable to identify any study of pediatric patients with primary brain tumors not of intrinsic anterior visual pathway origin and their effects on VR-QOL.

One limitation of this project is that as a quality improvement project its findings are not generalizable to general pediatric population. However, we hope that this project leads to future prospective evaluations of these at risk children to prevent vision loss and loss of QOL as many may not see an ophthalmologist. Indeed, in an institution-based study including 141 pediatric patients with brain tumors (including intrinsic visual pathway lesions) only 48% were referred for ophthalmologic evaluation; 79% of patients with neoplasms involving the temporal, parietal, and occipital lobes were not referred [5]. A six-month time-point following initial diagnosis was used to minimize effects from any acute intervention and its sequelae from acutely influencing QOL scores but it is unlikely that all patients were at the same stage of therapy due to the wide variety of lesions studied. Another limitation of this study is the lack of an intrinsic control group of normal children with whom to directly compare our QOL results. For the youngest patients who were not able to participate in formal visual acuity testing that would produce a numerical result, use of the central, steady, and maintained method may lead to overestimation of vision for these patients. Therefore, younger children may have visual impairment that we were unable to document due to age and cooperation. We were also limited in our analysis of the younger cohort of children due to the small numbers under the age of eight, and therefore of the number of patients completing the CVFQ. Some of this limitation may be due to later presentation to ophthalmology evaluation of children who do not complain of visual changes. We were unable to compare the VR-QOL outcomes of those under the age of eight to older children due to the use of two different VR-QOL instruments; no single instrument was available to evaluate the entire age range at the time of this project. Additionally, our small numbers of patients in each subgroup for tumor type and treatment modalities likely limited our ability to detect differences in these groups as this was a quality improvement project and therefore not designed with *a priori* power calculation of formal analysis plans typical of a research project. Future studies will need to be evaluated for numbers needed to evaluate to detect small differences between these groups. Finally, another limitation is the lack of a VR-QOL questionnaire that encompasses all ages.

In our quality improvement project, we found that children with primary brain tumors, excluding intrinsic tumors of the visual pathways, had decreased VR-QOL when compared with normal children in prior studies [11]. VR-QOL scores were found to correlate with VA, with worse VA and legal blindness associated with lower VR-QOL scores. This work stresses the importance of obtaining systematic ophthalmic examinations on children with brain tumors, due to the high rates of visual morbidity, as well as effects on QOL. We recommend at our site that all children with primary brain tumors undergo systematic ophthalmic examinations. This quality improvement project allowed for more streamlined access to ophthalmologic evaluations and revealed that vision affects quality of life for the neuro-oncology patient beyond what is captured in HR-QOL evaluations. Detailed ophthalmic examinations combined with VR-QOL assessments can be used to individualize treatment, prioritize referrals for low-vision assessments and visual aids, and complete a portion of the evaluation necessary in management of these individuals in the multi-disciplinary team. The goal should be the prevention of vision loss and better outcomes through earlier interventions from the ophthalmic perspective, as well as from a multi-disciplinary team perspective in managing the patient as a whole.

## Figures and Tables

**Table 1 –:** Demographics and Tumor Type

Demographics	n (%)
Male	48 (62%)
Female	29 (38%)
Age (years)	8 (IQR 4–11)
Craniopharyngioma	16 (21%)
Astrocytoma	15 (20%)
Medulloblastoma	10 (13%)
Ependymoma	8 (10%)
Glioma	6 (8%)
DNET	4 (5%)
Pituitary adenoma	3 (4%)
Glioblastoma	3 (4%)
Teratoma	2 (3%)
Meningioma	2 (3%)
Ganglioglioma	1 (2%)
PNET	1 (2%)
Other Tumor	6 (8%)

DNET = dysembryoplastic neuroepithelial tumor, PNET = primitive neuro-ectodermal tumor

**Table 2 –:** Rates of ophthalmologic abnormality, attributed causes, and vision-related quality of life score

Ophthalmologic abnormality	Attributed cause	Number of patients n (%)	Eye-Q score (p-value)
Visually impaired		44 (57%)	3.25 (p=0.05)
Legally blind		7 (9%)	0.7 (p<0.001)
Decreased visual acuity	Total	22 (29%)	p=0.033
	Tumor or treatment involvement	8 (36%)	3.32
	Previous papilledema	2 (9%)	1.2
	Combined tumor, treatment, previous papilledema	4 (18%)	3.25
	Amblyopia	6 (27%)	0.4
	Corneal complications	2 (9%)	2.65
Abnormal visual fields	Total	41 (53%)	p=0.078
	Tumor or treatment involvement	28 (61%)	3.35
	Previous papilledema	10 (24%)	3.2
	Combined tumor, treatment, previous papilledema	3 (7%)	1.07
Strabismus	Total	25 (32%)	p=0.2
	Tumor or treatment involvement	13 (52%)	3.03
	Sensory	9 (36%)	2.56
	Decompensated childhood strabismus	2 (8%)	3.78
	Other	1 (4%)	3.55
Optic nerve atrophy (either eye)	Total	29 (38%)	p=0.03
	Tumor or treatment involvement	18 (62%)	3.55
	Previous papilledema	7 (24%)	3.3
	Combined tumor, treatment, previous papilledema	4 (13%)	0.4

## Appendix 1: Additional questions asked of participants and their parents:

1. Do you require special assistance at school, such as an independent education plan (IEP) or 504 plan?
2. Do you wear glasses? Do you require additional low vision assistance, such as brail, a cane, or magnifier? If so, please specify.
